# Supplementary material for: Research on the Electron Structure and Antimicrobial Properties of Mandelic Acid and Its Alkali Metal Salts
Source: Int J Mol Sci. 2023 Feb 4;24(4):3078. doi: 10.3390/ijms24043078 (PMC9962254; doi:10.3390/ijms24043078)
Supplement: Supplementary file 1 [file ijms-24-03078-s001.zip › ijms-2177896-supplementary.pdf]

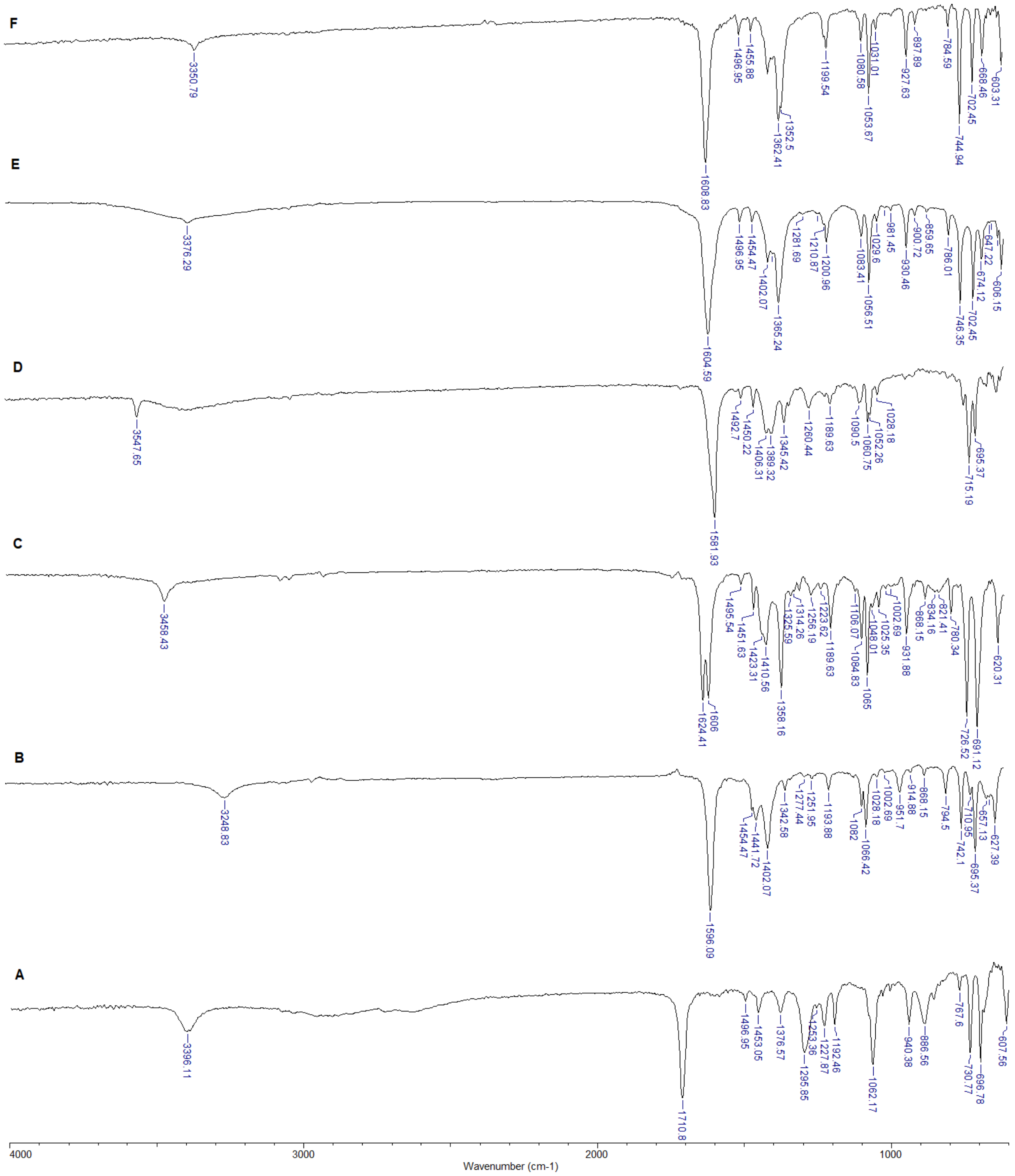

Figure S1. FTIR ATR spectra for mandelic acid (A) and lithium (B), sodium (C), potassium (D), rubidium (E), cesium mandelate (F)

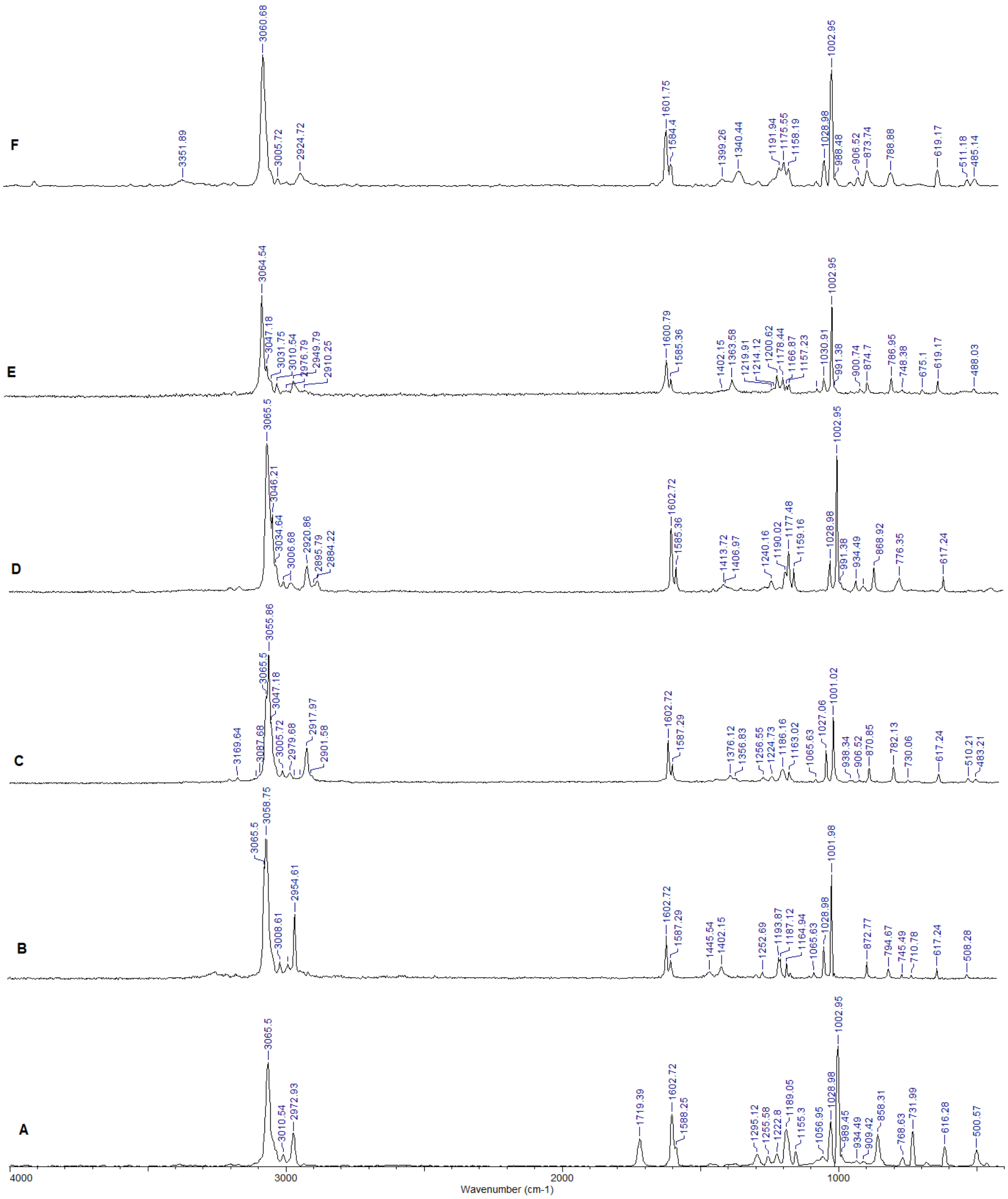

Figure S2. Raman spectra for mandelic acid (A) and lithium (B), sodium (C), potassium (D), rubidium (E), cesium mandelate (F)
